# Antitumor Activity of 6-(cyclohexylamino)-1, 3-dimethyl-5(2-pyridyl)furo[2,3-d]pyrimidine-2,4(1H,3H)-dione and Its Ti(IV), Zn(II), Fe(III), and Pd(II) Complexes on K562 and Jurkat Cell Lines

**DOI:** 10.1155/2008/501021

**Published:** 2009-01-26

**Authors:** Fahmideh Shabani, Shahriar Ghammamy, Khayroallah Mehrani, Mohammad Bagher Teimouri, Masoud Soleimani, Saeid Kaviani

**Affiliations:** ^1^Department of Chemistry, Islamic Azad University, Young Researchers club, Ardabil Branch, 56157-31567 Ardabil, Iran; ^2^Department of Chemistry, Faculty of Science, Imam Khomeini International University, 34149-16818 Ghazvin, Iran; ^3^Petrochemical Department, Iran Polymer and Petrochemical Institute, P.O. Box 14965-115, Tehran, Iran; ^4^Department of Hematology, Faculty of Medicine, Tarbiat Modarres University, P.O. Box 14115-318, Tehran, Iran

## Abstract

(6-(cyclohexylamino)-1,3-dimethyl-5(2-pyridyl)furo[2,3-d]pyrimidine-2,4(1H,3H)-dione) abbreviated as CDP was synthesized and characterized. Ti(IV), Zn(II), Fe(III), and Pd(II) metal complexes of this ligand are prepared by the reaction of salts of Ti(IV), Zn(II), Fe(III), and Pd(II) with CDP in acetonitrile. Characterization of the ligand and its complexes was made by microanalyses, FT-IR, ^1^H NMR, ^13^C NMR, and UV-Visible spectroscopy. All complexes were characterized by several techniques using elemental analysis (C, H, N), FT-IR, electronic spectra, and molar conductance measurements. The elemental analysis data suggest the stoichiometry to be 1:1 [M:L] ratio formation. The molar conductance measurements reveal the presence of 1:1 electrolytic nature complexes. These new complexes showed excellent antitumor activity against two kinds of cancer cells that are K562 (human chronic myeloid leukemia) cells and Jurkat (human T lymphocyte carcinoma) cells.

## 1. INTRODUCTION

Nitrogen-containing ligands such as Schiff bases and their metal complexes played an important role in the development of coordination chemistry, resulting in an enormous number of publications, ranging from pure synthetic work to physicochemical [1] and biochemically relevant studies of metal complexes [2–6] and found wide range of applications. Other kinds of nitrogen-containing ligands are well-known pyrimidine systems such as purine analogues that exhibit a wide range of biological activities. Fused pyrimidine compounds are valued not only for their rich and varied chemistry, but also for many important biological properties. Among them, the furopyrimidine ring system, because of a formal isoelectronic relationship with purine, is of special biological interest. It has numerous pharmacological and agrochemical applications, namely, antimalarials, antifolates, and antivirus, as well as potential radiation protection agents. Recently, some furopyrimidines were shown to be potent vascular endothelial growth factor receptor2 (VEGFR2) and epidermal growth factor receptor (EGFR) inhibitors. Because of the importance of furo[2,3-d]pyrimidine derivatives, several methodologies for synthesizing them have already been developed. However, many of the synthetic protocols reported so far prolonged reaction times, harsh reaction suffer from disadvantages, such as relying on multistep reactions, needing anhydrous conditions, low yields, use of metal-containing reagents, and special instruments or starting materials. Therefore, the development of new and efficient methods for the preparation of furo[2,3-d]pyrimidine derivatives is still strongly desirable [7].

Pyrimidines represent a very interesting class of compounds because of their wide applications in pharmaceutical, phytosanitary, analytical, and industrial aspects, for example, as antibacterial, fungicide [8], anti-inflammatory, antihelmintics, antitubercular, anti-HIV, antidegenerative and hypothermic activities [8], and herbicides [9], and have biological activities [10–14].

It has long been known that metal ions involve in biological processes of life and have been subject of interest. The modes of action of these metal ions are often complex but are believed to involve bonding to the heteroatoms of the heterocyclic residues of biological molecules, that is, proteins, enzymes, nucleic acids, and so forth [15].

From these points of view, it is interesting to study different types of transition metal complexes of these biologically active ligands. In this paper, the synthesis, characterization, and antitumor properties of a number of the first row transition metal complexes with one of the above ligands have been studied.

## 2. MATERIALS AND METHODS

### 2.1. Chemicals and reagents

*N*,*N*′-dimethylbarbituric acid, 2-pyridinecarbaldehyde, titanium(VI) tetra fluoride, zinc(II) acetate dihydrate, iron(III) chloride hexahydrate, and palladium chlorides were Merck chemicals (Darmstadt, Germany) and were used without further purification. Organic solvents were reagent grade. Electronic spectra were recorded by Camspec UV-Visible spectrophotometer model Wpa bio Wave S2 100. The IR spectra were recorded using FT-IR Bruker Tensor 27 spectrometer. ^1^H-NMR and ^13^C-NMR were recorded on a Bruker AVANCE DRX 500 spectrometer. All the chemical shifts are quoted in ppm using the high-frequency positive convention; ^1^H and ^13^C-NMR spectra were referenced to external SiMe_4_. The percent composition of elements was obtained from the Microanalytical Laboratories, Department of Chemistry, OIRC, Tehran.

### 2.2. Cell culture

The human chronic myeloid leukemia—K562 cell line—and the human T lymphocyte carcinoma-Jurkat cell line, used for treatment with the drugs, were provided. K562 and Jurkat cells were grown at 37°C in an atmosphere containing 5% CO_2_, with RPMI-1640 Medium HEPES Modification with L-glutamine and 25 mM HEPES (Sigma-Aldrich Chemie GmbH, Germany) supplemented with 10% heat-inactivated fetal bovine serum (FBS) (Gibco, Carlsbad, Calif, USA), 2.7% sodium bicarbonate, and 500 mg/L ampicillin.

## 3. EXPERIMENTAL

### 3.1. Synthesis of the CDP ligand

**Figure 1:**
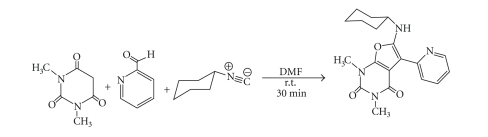
Synthesis route of CDP ligand.

To a solution of *N*, *N*′-dimethylbarbituric acid (0.78 g, 5.0 mmol) and 2-pyridinecarbaldehyde (0.54 g, 5.0 mmol) in DMF (3 mL) in a screw-capped vial was added cyclohexyl isocyanide (0.55 g, 5.0 mmol) via a syringe and was shaken for 1 minute. The reaction mixture was then kept for about 30 minutes at room temperature (25°C) and the completion of reaction was confirmed by TLC (EtOAc-hexane 1:2). Then, the resulting crystals were filtered and washed with diethyl ether (20 mL) to yield as light pink crystals (1.42 g, 80%). The dried product thus obtained showed a single spot on TLC and was pure enough for all analytical purposes [7] (see Figure 1).

#### 3.1.1. Analysis of CDP ligand

Yield, 80%. Mp 135.2–137.5°C; Anal. Calcd. C_19_H_21_N_4_O_3_: C, 64.58; H, 5.94; N, 15.86. Found: C, 64.92; H, 6.29; N, 16.08. ^1^H NMR (CDCl_3_): 4.40(1H, d, NH), 8.40 and 7.60(1H, d, pyridine), 7.55 and 7.26(1H, d, pyridine), 3.61(3H, s, NCH_3_), 3.45(3H, s, NCH_3_), 1.38(2H, m, cyclohexan), 1.63(2H, m, cyclohexan), 2.01(1H, m, cyclohexan); ^13^C NMR (CDCl_3_): 158.63(C_4_), 154.51(NCON), 153.42(C_2_), 136.27(C_6_), 122.3(C_3_), 149.9, 149.4, 147.2, and 118.4(pyridine), 52.34, 33.92, and 24.49(cyclohexan). IR (KBr, cm^−1^): 3276 w, 1664 w, 1593 w, 1449 m, 1266 w, 1120 w, 610, and 742 s. UV-vis (MeCN): *λ*_max_260 nm (*ε* 120), 336 nm (*ε* 110).

### 3.2. Synthesis of the metal complexes: general method

A solution of metal salt dissolved in acetonitrile was added gradually to a stirred acetonitrile solution of the ligand (CDP), in the molar ratio 1:1 (metal:ligand). The reaction mixture was further stirred for 2–4 hours to ensure the completion and precipitation of the formed complexes. The precipitated solid complexes were filtered and washed several times with 50% (v/v) ethanol/water to remove any traces of the unreacted starting materials. Finally, the complexes were washed with diethyl ether and dried in vacuum desiccators over anhydrous CaCl_2_.

#### 3.2.1. Analysis of Ti(C_19_H_20_N_4_O_3_)F_4_

Yield, 85%. Anal. Calcd. Ti(C_19_H_20_N_4_O_3_)F_4_: C, 47.89; H, 4.20; N, 11.76. Found: C, 48.2; H, 4.37; N, 12.1. ^1^H NMR (DMSO): 9(1H, d, pyridine), 8.30(1H, d, pyridine), 7.1(1H, d, pyridine), 3.53(3H, s, NCH_3_), 3.01(3H, s, NCH_3_), 1.08–2.5(2H and 1H, m, cyclohexan); IR (KBr, cm^−1^): 1689 s, 1625 m, 1453 m, 1246 w, 1153 w, 774 s, 667 w, and 603 s. UV-vis (MeCN): *λ*_max_377 nm (*ε* 54), 497 nm (*ε* 28).

#### 3.2.2. Analysis of Zn(C_19_H_20_N_4_O_3_)(OAC)_2_

Yield, 60%. Anal. Calcd. Zn(C_19_H_20_N_4_O_3_)(OAC)_2_: C, 42.57; H, 3.73; N, 10.45. Found: C, 43.5; H, 3.86; N, 10.82. ^1^H NMR (DMSO): 8.30(1H, d, pyridine), 8.06(1H, d, pyridine), 7.5(1H, d, pyridine), 3.3(3H, s, NCH_3_), 3(3H, s, NCH_3_), 1.2–2.35(2H and 1H, m, cyclohexan); IR (KBr, cm^−1^): 1691 s, 1625 m,1439 w, 1246 w, 1158 w, and 425 s. UV-vis (MeCN): *λ*_max_262 nm (*ε* 110), 302 nm (*ε* 100), 344 nm (*ε* 78), and 415 nm (*ε* 60).

#### 3.2.3. Analysis of Fe(C_19_H_20_N_4_O_3_)Cl_3_

Yield, 75%. Anal. Calcd. Fe(C_19_H_20_N_4_O_3_)Cl_3_: C, 44.32; H, 3.88; N, 10.88. Found: C, 44.86; H, 4.18; N, 11.38. ^1^H NMR (DMSO): 9.67(1H, d, pyridine), 9.02(1H, d, pyridine), 7.95(1H, d, pyridine), 3.7(3H, s, NCH_3_), 3.1(3H, s, NCH_3_), 1.5–2.8(2H and 1H, m, cyclohexan); IR (KBr, cm^−1^): 1600 w, 1546 w, 1444 w, 1154 s, 514 m, and 599 s. UV-vis (MeCN): *λ*_max_257 nm (*ε* 280), 314 nm (*ε* 156), 363 nm (*ε* 126), 440 nm (*ε* 60), and 494 nm (*ε* 28).

#### 3.2.4. Analysis of Pd(C_19_H_20_N_4_O_3_)Cl_2_

Yield, 88%. Anal. Calcd. Fe(C_19_H_20_N_4_O_3_)Cl_3_: C, 43.07; H, 3.77; N, 10.57. Found: C, 43.45; H, 3.95; N, 10.89. ^1^H NMR (DMSO): 8.31(1H, d, pyridine), 7.55(1H, d, pyridine), 7.37(1H, d, pyridine), 2.55(3H, s, NCH_3_), 2.5(3H, s, NCH_3_), 1.18–2.07(2H and 1H, m, cyclohexan); IR (KBr, cm^−1^): 1649 s, 1546 m, 1467 m, 1266 m, 1142 m, and 495 m. UV-vis (MeCN): *λ*_max_261 nm (*ε* 220), 307 nm (*ε* 130), 442 nm (*ε* 118), and 660 nm (*ε* 75).

### 3.3. Cytotoxicity studies

CDP ligand and Ti(C_19_H_20_N_4_O_3_)F_4_, Zn(C_19_H_20_N_4_O_3_) (OAC)_2_, Fe(C_19_H_20_N_4_O_3_)Cl_3_, and Pd(C_19_H_20_N_4_O_3_)Cl_2_ complexes are five compounds which were assayed for cytotoxicity in vitro against K562 (human chronic myeloid leukemia) cells and Jurkat (human T lymphocyte carcinoma) cells.

The two cell lines were provided by the Pasteur Institute in Iran. The procedure for cytotoxicity studies was similar to that reported earlier [16]. Briefly, in order to calculate the concentration of each drug that produces a 50% inhibition of cell growth (IC_50_), 190 mL of cell suspension 4 × 10^5^ cell/mL) was exposed to various concentrations of ligand and complexes dissolved in sterile DMSO. The final concentration of DMSO in the growth medium was 2% (v/v) or lower, concentrations without effect on cell replication [17, 18].

**Figure 2:**
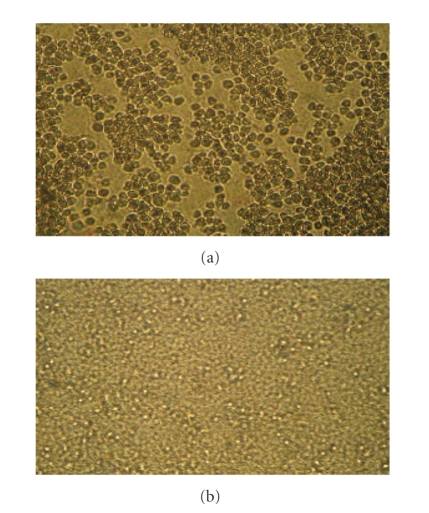
(a) Tumor cell after 72 h without Pd(C_19_H_20_N_4_O_3_)Cl_2_ compound. (b) tumor cell after 72 h with Pd(C_19_H_20_N_4_O_3_)Cl_2_ compound.

After the incubation periods 72 hours for all cell lines, the cell concentrations were determined both in control and in drug-treated cultures. All experiments were done for six times (see Figure 2).

## 4. RESULTS AND DISCUSSION

### 4.1. Preparation for ligand, CDP, and Ti(IV), Zn(II), Fe(III), and Pd(II) complexes

The reaction of Ti(IV), Zn(II), Fe(III), and Pd(II) salts with the ligand, CDP, results in the formation of [ML] for M = Ti(IV), Zn(II), Fe(III), and Pd(II). All complexes are quite stable and could be stored without any appreciable change. All complexes were characterized by several techniques using elemental analyze (C, H, N), FT-IR, electronic spectra, and molar conductance measurements. The elemental analysis data suggest the stoichiometry to be 1:1 [M:L] ratio formation. The molar conductance measurements reveal the presence of 1:1 electrolytic nature complexes. The complexes Ti(C_19_H_20_N_4_O_3_)F_4_, Zn(C_19_H_20_N_4_O_3_)(OAC)_2_, and Pd(C_19_H_20_N_4_O_3_)Cl_2_ do not have sharp melting points but decompose above 237°C, 290°C, and 331°C, respectively, but Fe(C_19_H_20_N_4_O_3_)Cl_3_ complex has 153°C–155°C melting point. They are insoluble in common organic solvents, such as ethanol, methanol, chloroform, or acetone; however, they are soluble in DMSO and DMF. Their structures were characterized by elemental analysis, ^1^HNMR and IR. Their elemental analyses are in accord with their proposed formula. The spectral data of the complexes have good relationship with the literature data.

### 4.2. Cytotoxicity assays in vitro

CDP ligand and Ti(C_19_H_20_N_4_O_3_)F_4_, Zn(C_19_H_20_N_4_O_3_) (OAC)_2_, Fe(C_19_H_20_N_4_O_3_)Cl_3_, and Pd(C_19_H_20_N_4_O_3_)Cl_2_ complexes have been tested against two human cancer cell lines: K562 and Jurkat. The IC_50_ cytotoxicity values of the complexes were compared to those found for the starting organic bases as well as for some of the anticancer agents used nowadays, that are cisplatin and oxaplatin compounds [19].

The general method used for testing on antitumor properties of these compounds is the standard testing method that has been previously described in greater detail.

After preincubation lasting for 12 hours at 37°C in a 5% CO_2_ atmosphere and 100% humidity, the tested compounds in the concentration ranges of 0.1–250 *μ*M for CDP, of 0.1–150 *μ*M for Ti(C_19_H_20_N_4_O_3_)F_4_, of 0.1–100 *μ*M for Zn(C_19_H_20_N_4_O_3_)(OAC)_2_, of 0.1–200 *μ*M for Fe(C_19_H_20_N_4_O_3_)Cl_3_, and of 0.1–97 *μ*M for Pd(C_19_H_20_N_4_O_3_)Cl_2_ were added. The incubation lasted for 72 hours and at the end of this period IC_90_ and IC_50_ of the dead cells and live cells were measured by trypan blue. The mechanism by which these complexes act as antitumor agents is apoptosis. IC_90_ and IC_50_ values that are the compounds concentrations lethal for 90% and 50% of the tumor cells were determined both in control and in compounds concentrations lethal for both in compounds-treated cultures. The compounds were first dissolved in DMSO and then filtrated. The corresponding 50% and 90% inhibitory doses (IC50 and IC90) values are shown in Table 1.

## 5. CONCLUSION

It is clear from the above discussion that Ti(IV), Zn(II), Fe(III) and Pd(II) complexes and CDP ligand offer a new outlook for chemotherapy. The results of antitumor activity show that the metal complexes exhibit antitumor properties and it is important to note that they show enhanced inhibitory activity compared to the parent ligand. The mechanism by which these complexes act as antitumor agents is apoptosis. It has also been proposed that concentration plays a vital role in increasing the degree of inhabitation.

## Figures and Tables

**Table 1:** 72-hour IC_50_ and IC_90_ values (*μ*M) obtained for CDP and three CDP complexes.

Complexes	IC_50_ for cell line		IC_90_ for cell line	
	K562	Jurkat	K562	Jurkat
CDP	>110	>110	—	—
Ti(C_19_H_20_N_4_O_3_)F_4_	>70	>50	>100	>100
Zn(C_19_H_20_N_4_O_3_)(OAC)_2_	>100	>100	>150	>150
Fe(C_19_H_20_N_4_O_3_)Cl_3_	>45	>40	>120	>120
Pd(C_19_H_20_N_4_O_3_)Cl_2_	>36	>33	>85	>80
